# Characterization of BRAF mutation in patients older than 45 years with well-differentiated thyroid carcinoma

**DOI:** 10.1016/j.bjorl.2020.07.007

**Published:** 2020-09-12

**Authors:** Luis Rene Quiroa Barreno, Julia Bette Homem de Mello, Mateus Camargo Barros-Filho, Ana Lucia Francisco, Thiago Celestino Chulam, Clovis Antonio Lopes Pinto, Joao Gonçalves-Filho, Luiz Paulo Kowalski

**Affiliations:** a A.C. Camargo Cancer Center, Departamento de Cirurgia de Cabeça e Pescoço e Otorrinolaringologia, São Paulo, SP, Brazil; b Instituto Nacional do Câncer (INCA), Programa de Carcinogênese Molecular, Rio de Janeiro, RJ, Brazil; c A.C. Camargo Cancer Center, Departamento de Patologia, São Paulo, SP, Brazil; d Universidade de São Paulo, Faculdade de Medicina, São Paulo, SP, Brazil

**Keywords:** Thyroid cancer, Papillary carcinoma, BRAF mutation, Survival, Recurrence, Prognosis

## Abstract

**Introduction:**

Papillary thyroid carcinoma is the most frequent endocrine neoplasia and its incidence has tripled over the past 35 years. Although papillary thyroid carcinoma carries a good prognosis, 10%−30% of patients still develop recurrence and metastasis. Some clinical and genetic features are associated with worse prognosis. The most frequent mutation is the BRAF p.V600E, which has been associated with many clinical features of poor prognosis. However, many studies have produced controversial results without any association between BRAF mutation and clinicopathological features of poor prognosis.

**Objective:**

Since the prognostic value of BRAF mutations remains controversial, this study aims to investigate the importance of this mutation in therapeutic decisions for papillary thyroid carcinoma.

**Methods:**

Therefore, we evaluated whether the presence of BRAF mutation is associated with features of poor prognosis in 85 patients with papillary thyroid carcinoma older than 45 years treated at A.C. Camargo Cancer Center, from 1980 to 2007. BRAF mutation was evaluated by pyrosequencing. Statistical analysis was performed using SPSS.

**Results:**

The mean age of patients was 54 years (range: 45 − 77 years), 73 were women (85.8%) and 12 were men (14.2%). Among them, 39 cases (45.9%) presented extrathyroidal extension and 11 cases had recurrent disease. BRAF mutation was detected in 57 (67%) patients. No significant association was observed between BRAF mutation and gender (*p*  = 0.743), age (*p*  = 0.236), N-stage (*p*  = 0.423), vascular and perineural infiltration (*p*  = 0.085 or multifocality (*p*  = 1.0). Although not statistically significant, the majority of patients with recurrent disease were BRAF positive (9 out of 11) (*p*  = 0.325). Patients affected by BRAF mutation are associated with tumors larger than 1 cm (*p*  = 0.034) and with extrathyroidal extension (*p*  = 0.033).

**Conclusion:**

Although BRAF testing is widely available, there are no consistent data to support improvement in outcomes from incorporating it into therapeutic decision for thyroid cancer.

## Introduction

Well-Differentiated Thyroid Carcinoma (DTC) is the most common endocrine malignancy; the incidence has triple over the last 35 years affecting more than 50.000 people in USA in 2018.^1^ The majority of thyroid carcinomas are derived from thyroid follicular cells that give rise to Papillary (PTC) and Follicular (FTC) carcinoma subtypes.^2^ PTC represents 80%−85% of all thyroid carcinomas, and a few risk factors have been associated with disease development, including radiation exposure, low consumption of iodine and family history of thyroid carcinoma.^2^, ^3^

In general, PTC has a good prognosis with a 5 year survival rate over 90%, however, some patients are unresponsive to treatment and more accurate prognostic markers are not well established in clinical routine.^4^ There is a risk of recurrence ranging from 5%−30%, depending on the disease stage at diagnosis.^5^ Currently, critical factors in outcome of patients with PTC are age at diagnosis (> 55 years) and presence of extrathyroidal extension. Additional factors include tumor size, histological type, presence of metastases, and incomplete tumor resection.^4^, ^5^ Besides clinical and pathological factors, genetic alterations have also been evaluated in PTC, the most important molecular variant is the BRAF p.V600E mutation.^6^

BRAF mutations frequency ranges from 29%–87% in population (mean of 45%).^7^, ^8^ Although more than 40 mutations have been identified in BRAF, the most frequent hot spot, accounting for over 90% of all BRAF mutations, is T1799A BRAF transversion nucleotide originating a V600E mutant protein with constitutive activation of BRAF kinase.^9^, ^10^ BRAF is a potent activator of mitogen-activated protein kinase (MAPK) pathway, which has a major role in regulation of cell growth and division by blocking apoptosis and regulating proliferation and invasion.^11^, ^12^

Several studies have reported a correlation of BRAF V600E with clinical and pathological features of more aggressive PTC, such as distant or nodal metastases, larger tumor size and advanced stage.^11^, ^12^ The presence of the BRAF p.V600E mutation has been included to the latest recommendations of the American Thyroid Association (ATA)^13^ as a factor to be consider in stratification for the risk of a poor clinical course in PTC.

However, a series of studies observed no association between BRAF mutation and clinicopathological markers of poor prognosis.^14^, ^15^ An overview of literature indicates that the clinical data linking BRAF mutation and DTC outcomes are not very consistent, with many controversial results reported.^14^, ^16^, ^17^ Since, the prognostic significance of BRAF mutations remains a topic of debate with conflicting reports, in this study we aim to evaluate if the presence of BRAF mutation is a prognostic factor in patients with papillary thyroid carcinoma older than 45 years old.

## Methods

### Patients

This was a retrospective study with a review of patients submitted to total thyroidectomy between January 1980 and December 2007 at the Head and Neck and Otorhinolaryngology Department of AC Camargo Cancer Center (Brazil). We enrolled in this study 85 PTC samples available for DNA extraction from patients with ≥ 45 years. The study was approved by the Institutional Human Research Ethics Committee (Protocol # 1541/11).

### Clinical and histopathological characterization

Clinical data was collected for each patient, including age, sex, risk factors for thyroid cancer and followup (postoperative scintigraphy, serum thyroglobulin ultrasound, or CT scan). Detection of persistent and recurrent disease was evaluated by ultrasound, serum thyroglobulin measurement in patients without anti-Tg antibodies (with or without rhTSH stimulation), 131I scan, and FNAB according to the ATA guidelines for follow-up (i.e., using the 6-month cutoff).^13^ Histological classification of samples was revised by an experienced pathologist using blinded interpretation and pathological features were assessed, as histological variant, vascular invasion, multifocality and TNM staging.^18^

### Tissue processing and DNA extraction

Surgical specimens were properly collected and stored in AC Camargo Cancer Center Tumor Bank, following all legal and ethical requirements. Fresh frozen samples from papillary thyroid carcinomas were obtained from 85 patients undergoing surgical resection. Samples were manually microdissected to achieve at least 70% malignant representation and DNA was extracted using a standard phenol/chloroform-based method. DNA integrity and concentration were verified in gel agarose and NanoDrop (ND-1000 Spectrophotometer v. 3.0.1, Labtrade).

### BRAF V600E detection by pyrosequencing

Initially, the region of interest was amplified by PCR (codon 600 of BRAF) with 20 ng of DNA and 0.2 μM of labeled primers (Epigendx, Worcester MA, England) (assay ADS871) and PCR MasterMix (Qiagen). Controls without template, a positive (colon cancer cell linage WiDr) and negative controls (human DNA non-methylated from Epitech, Qiagen) were included in all runs. In order to confirm successful amplifications, the PCR products were electrophoresed in a 1% agarose gel. Pyrosequencing was carried out according to manufactures’ recommendations in PyroMark Q96 ID system (Qiagen, Valencia, USA). Exchange of nucleotide-T for A in codon 600 in more than 10% of alleles were assumed positive for mutation in BRAF.

### Statistical analysis

Association between BRAF mutation and categorical variables was tested with the X2 test or Fisher exact test as appropriate. The Student *t*-test was used to evaluate the association with continuous variables with normal distribution, whereas the Mann-Whitney *U* test was used for abnormal distributed continuous variables. Disease free survival curves were analyzed using the Kaplan-Meier method, assessing significance of the statistical difference between BRAF-positive and BRAF-negative patients with the log-rank test. Finally, Cox regression analysis of recurrence-free survival time was performed, including BRAF status and all variables associated with recurrence at the univariate analysis. Statistical significance was considered for *p* < 0.05, and confidence intervals were set at 95%. Statistical analysis was performed using SPSS (v. 21.0; SPSS, Chicago, IL, USA) and Graphpad Prism (v. 5.0; GraphPad Software Inc., La Jolla, CA, USA) software.

## Results

### Clinical data

Among the 85 patients included on this study, 73 were women (85.8%) and 12 were men (14.2%). The mean age was 54 years (range, 45 − 77 years) and 29 (34%) patients were older than 55 years. All tumors evaluated were papillary thyroid carcinoma: histological variant types were revised by the pathologist and 59 samples were classified as classical (69.4%), 19 were follicular (22.3%) and 6 were rare variant subtypes (8.3%). Extrathyroidal extension was present in 39 cases (45.9%), and vascular and perineural infiltration were detected in seven patients (8.2%). The majority of cases presented single nodules (53 of 85, 62.4%) and 42 (49.4%) patients had thyroid nodules smaller than 1 cm. Median followup period was 92 months.

### BRAF mutation and association with clinical data

BRAF mutation was detected in 57 (67%) of 85 patients, and the patients were classified in two groups according to BRAF V600E status (BRAF positive vs. negative) (Table 1). Gender was not associated with BRAF status (*p* =  0.743), neither age at diagnosis (*p* =  0.236). No significant association was observed between BRAF mutation and histologic variant (*p* =  0.075), N-stage (*p* =  0.423), vascular and perineural infiltration (*p* =  0.085), multifocality (*p* =  1.0) and lymph nodal metastasis (*p* =  0.423).

**Table 1:** Clinical and pathological data of 85 patients and association with Braf^V600E^ mutation.

Variable	Category	BRAF^V600E^ (%)		*p*-value
		Negative	Positive	
Gender	Male	3 (25)	9 (75)	0.743
	Female	25 (34.2)	48 (65.8)	
Age (years)	≤ 55	21 (37.5)	35 (62.5)	0.236
	> 55	7 (24.1)	22 (75.9)	
Tumour size	≤ 1 cm	9 (21,4)	33 (78,6)	0.037
	> 1 cm	19 (44,2)	24 (55,8)	
Lymph node status	pN0	23 (35,9)	41 (64,1)	0.423
	pN1	5 (23,8)	16 (76,2)	
Histological variant	Papillary carcinoma	15 (25,4)	44 (74,6)	0.075
	Follicular carcinoma	10 (52,6)	9 (47,4)	
	Rare	3 (42,9)	4 (57,1)	
Multifocality	No	18 (34)	35 (66)	1.0
	Yes	10 (32,3)	21 (67,7)	
Extrathyroidal Extension	No	19 (44,2)	24 (55,8)	0,033
	Yes	8 (20,5)	31 (79,5)	
Vascular/ Perineural Infiltration	No	28 (36,4)	49 (63,6)	0,089
	Yes	0 (0)	7 (100)	
Recurrence	No	26 (35,1)	48 (64,9)	0,325
	Yes	2 (18,2)	9 (81,8)	

**Figure 1:**
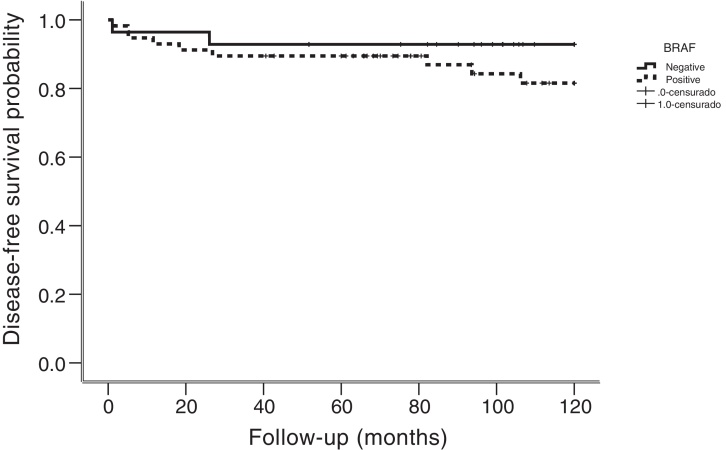
Disease Specific Survival according with the presence of BRAF mutation.

Patients affected by BRAF mutation are associated with tumors of more than 1 cm (*p* =  0.034) and with extrathyroidal extension (*p* =  0.033). Recurrence of disease was detected in 11 of 85 (13%) patients, nine of them were positive for BRAF mutation. Recurrences were locally and regional in nine patients, whereas two had distant disease. The median followup of patients with recurrence was 34 months. All recurrences had pathologic confirmation (fine needle aspiration biopsy or surgery) or combined imaging and strong biochemical evidence. Although the majority of patients with recurrence were BRAF positive, no statistical significance was found (*p* =  0.325). Mean time to recurrence was 18 months (range, 1−106 months). Kaplan-Meier analysis for specific disease survival showed no statistically significant association with BRAF mutation (*p* =  0.272) (Fig. 1).

## Discussion

Well-differentiated thyroid carcinomas derived from follicular epithelium are the most frequent carcinoma of endocrinology system and the predominant histological type is the papillary carcinoma, corresponding to 80%−90% of all DTC.^19^ Papillary thyroid carcinomas are usually indolent with favorable long-term survival using currently established treatments. Ten year overall survival rates for these patients is nearly 90%, however 5%−10% may develop regional recurrence and 10%−15% distant metastases.^2^, ^20^ Some pathological features that are associated with worse prognosis include: larger tumors with extrathyroidal extension, distant metastasis, and cervical lymph node metastases. An additional prognostic molecular marker is the BRAF V600E mutation, which is the most common alteration in DTC.^6^ Many studies have demonstrated an association of BRAF mutation with poorer outcomes of PTC.^21^ In contrast, additional studies have not found any correlation of this mutation with worse prognosis of PTC.^15^, ^22^ Considering these controversial findings on the role of BRAF V600E in clinicopathological aggressiveness of PTC, we investigated the presence of this mutation in 85 PTC from patients older than 45 years and its association with clinical features of poor prognosis. BRAF mutation was detected in 67% of cases; this frequency is similar to previously described by Italian studies, which reported frequencies up to 66%.^23^ On the other hand, in the Japanese population the prevalence is lower, being observed in only 38% of patients, which is similar to the percentage in the USA (40%).^15^, ^24^

In order to investigate the potential prognostic value of BRAF mutation in Brazilian population, we associated the mutation status with several features of high recurrence risk, including age over 55 years, male gender, multifocality, extrathyroidal extension, histological type, vascular and lymphatic invasion, lymph node metastasis and distant metastasis. Several studies have reported an association between age and mortality rates according to BRAF mutation status.^4^, ^7^ Recently, a large study involving 2638 patients with DTC demonstrated a correlation between increasing age and mortality in BRAF positive patients.^25^ However, no correlation was observed in BRAF negative patients, suggesting that age is a strong risk factor only for positive BRAF mutation but not for wild-type DTC patients.^25^ Since the cut‐off age of 45 years was controversial and the age of 55 years was demonstrated to be more appropriate to classify high-risk patients,^5^ in this study we enrolled patients over 45 years and compared them with the group of < 55 years. However, we did not find any association of age (> 45 and < 55) and mortality risk in BRAF mutated patients; in concordance with our findings, some studies have not found any association either.^5^, ^22^ Regarding gender as a prognostic feature, we did not detect any association of BRAF status and gender, although a meta-analysis study conducted by Li et al. demonstrated association between BRAF mutation and male patients.^26^

In this study, we could observe a significant difference between T-stages and BRAF mutation status (*p*  = 0.037). BRAF mutations were more prevalent in tumors larger than 1 cm, this association was also described by Li 2012 and Kim 2012.^11^, ^26^ Comparison of lymph node metastasis and BRAF mutation did not reveal any correlation (*p* =  0.423). The lack of correlation between this prognostic feature and BRAF was also observed by other studies. Pelttari et al. reported that BRAF mutation was not significantly associated with lymph node metastasis, tumor size and distant metastasis at the time of disease presentation.^27^, ^28^ Furthermore, Koo et al. reported that only tumor size and the presence of lymphatic and vascular invasion have been shown to be predictors of lymph node metastasis independently of BRAF mutation.^28^ In this study, we did not detect any correlation between lymphatic and vascular invasion, lymph node involvement and BRAF mutation (*p* =  0.089, *p* =  0.433, respectively).

Extrathyroidal extension is an important feature of poor prognostic value for PTC and has been associated with increased risk of invasion into cervical structures, local recurrence, and persistence of tumor.^21^ A meta-analysis conducted by Liu et al. including 10,301 patients reported that tumors harboring BRAF mutation have an increased risk of extrathyroidal extension compared to BRAF negative cases.^21^ In addition, the majority of studies in the literature have reported higher frequency of extrathyroidal extension in patients BRAF mutated.^21^, ^29^ On this study, we also observed that majority of patients with extrathyroidal extension were BRAF positive (80%) (*p*  = 0.033), however, microscopic or macroscopic extension data are not available because this information was not evaluated at the time of surgical procedures. In addition, many studies have described association of BRAF mutation and multifocal tumors.^29^ However, in this study we could not detect any association between these features (*p* =  1.0).

Although the risk of recurrence in PTC is relatively low (5%−10%), it may affect the patient’s quality of life, requiring additional therapy, such as reoperation and high cumulative radioiodine dose. Several meta-analysis studies have found an association between BRAF mutation and recurrence.^21^, ^29^ A large multicenter study also reported that BRAF mutation is an independent prognostic factor for PTC recurrence.^27^ On this study, recurrence was observed in 11 patients and nine of them were BRAF positive. Although the majority of patients who had recurrence were mutated, we could not observe any significant association between these features (*p* =  0.325), probably due the limited number of patients.

## Conclusions

Although this study evaluated a small number of cases, our findings do not support the hypothesis of using the BRAF mutation as a prognostic factor for both disease recurrence and changing the surgical approach to perform prophylactic neck dissection in patients with positive mutation.

## Conflict of interest

The authors declare no conflicts of interest.
